# The spiritual health of veterans with a history of suicide ideation

**DOI:** 10.1080/21642850.2014.881260

**Published:** 2014-03-27

**Authors:** Marek S. Kopacz

**Affiliations:** ^a^ VISN 2 Center of Excellence for Suicide Prevention, US Department of Veterans Affairs, Canandaigua, NY, USA

**Keywords:** suicide, military, religion and spirituality

## Abstract

*Introduction*: In recent years, considerable empirical attention has been devoted to examining the increased risk of suicide observed in some Veteran populations. This has led to a renewed focus on developing novel support options which can be used to respond to Veterans in distress, reducing their risk of suicide. Spirituality and religion, however, have been largely absent from any public discourse related to suicide prevention, not least of all in Veteran populations. *Aim*: The aim of this cross-sectional study is to compare the self-rated spiritual health of Veterans with and without suicide ideation. Identifying differences which may exist between these two groups could highlight the relevance of spiritual well-being to Veteran suicide prevention efforts. *Materials and Methods*: Data were collected using pencil-and-paper surveys, called Spiritual Assessments, distributed within the general population of in- and outpatients at a U.S. Department of Veterans Affairs Medical Center. Using Likert-type scales, this study examines the self-rated spiritual health, spiritual devotion, and significance ascribed to spirituality in a sample of 5378 Veterans. Statistical analysis took place using chi-squared to examine differences in the distribution of responses between ideators and non-ideators. *Results*: Ideators significantly more often rated their spiritual health as worse than that of non-ideators. Even with similar levels of spiritual devotion or significance ascribed to spiritual life, ideators continued to significantly more often rate their spiritual health as worse than that of non-ideators. *Conclusion*: The results show that Veterans with suicide ideation more often rate their spiritual health as worse than that of Veterans without suicide ideation. This suggests that spiritual well-being may indeed be relevant to suicide prevention efforts in Veteran populations.

## Introduction

In recent years, considerable attention has been devoted to examining the increased risk of suicide observed in some Veteran populations (Bullman & Kang, 1996; Kang & Bullman, 2008, 2009; Mrnak-Meyer et al., 2011; Pietrzak, Russo, Ling, & Southwick, 2011). The number of Veterans who die by suicide is estimated at 18–22 per day, with Veterans accounting for 22% of all suicide completions in the USA (Kemp & Bossarte, 2012; National Violent Death Reporting System, 2012). Responding to this alarming trend remains a high priority for the Veterans Health Administration (VHA) (Department of Veterans Affairs, 2007). To this end, there has been a renewed focus on developing novel support options which can be used to respond to Veterans in distress, reducing their risk of suicide. This includes looking more closely at the protective potential of spirituality and religion.

Spiritual health is named as a protective factor in the *2012 National Strategy for Suicide Prevention* (U.S. Department of Health and Human Services, 2012). An enhanced sense of spiritual well-being may help maintain meaningful congruencies with one's environment and reduce feelings of dissonance in times of distress (Kopacz, Silver, & Bossarte, in press). The World Health Organization (2012) also names “religious beliefs that discourage suicide and support self-preservation” as protective against suicide. Religious involvement, in addition to contributing to a sense of spiritual well-being, is thought to also mitigate some of the behavioral, psychological, physical, and social risk factors associated with suicidal behavior (Anandarajah & Hight, 2001; Koenig, King, & Carson, 2012). Spirituality and religion, however, have been largely overlooked as part of organized suicide prevention efforts (Colucci & Martin, 2008).

Available research suggests that spirituality and religion remain an important part of the lives of many Veterans (Chang et al., 2012; House Committee on Veterans’ Affairs, 2012; LaPierre, 1994). One could reasonably assume that a spiritually minded suicide prevention program might then appeal to certain groups of Veterans. Upwards of 49% of Veterans report attending religious services at least once per month (Pew Social and Demographic Trends, 2011). Some Veterans also look to spirituality as a coping mechanism for dealing with stressful situations (Greenawalt et al., 2011; Mihaljević, Aukst-Margetić, Vuksan-Ćusa, Koić, & Milošević, 2012). This includes consulting pastoral care providers for the purposes of mental-health support (Besterman-Dahan, Gibbons, Barnett, & Hickling, 2012; Bonner et al., 2013; Iversen et al., 2010; Kirchner, Farmer, Shue, Blevins, & Sullivan, 2011; Sullivan, 2007). Religious counseling, as opposed to specialty mental-health services, may even be the preferred therapeutic option for some Veterans (Greenawalt et al., 2011).

The paucity of available literature makes applying spirituality and religion to Veteran suicide prevention efforts difficult. Only a small number of studies have directly examined spirituality in Veterans at increased risk of suicide (Kopacz, 2013; Mihaljević et al., 2011; Nađ, Marčinko, Vuksan-Æusa, Jakovljević, & Jakovljevic, 2008). Further, no published data are available for gauging the spiritual health, level of spiritual devotion, or significance ascribed to spirituality by at-risk Veterans. In effect, little is known of what differences may exist – in terms of spirituality, little is known of what differences may exist between Veterans at increased risk of suicide and those not otherwise at increased risk. Accordingly, the aim of this cross-sectional study is to compare the self-rated spiritual health of Veterans with and without suicide ideation. Identifying differences which may exist between these two groups stands to highlight the potential relevance of spiritual well-being to Veteran suicide prevention efforts.

## Materials and methods

### Study population and data collection

Data were collected from the general in- and outpatient population at the U.S. Department of Veterans Affairs Medical Center in Asheville, North Carolina. This medical center provides healthcare services through a number of clinical departments, such as hospice and palliative care, in- and outpatient surgery, spinal cord injury, extended care and rehabilitation, radiation, oncology, primary care, neurology, audiology, and mental health.

The variables analyzed as part of this study come from an 18-question, pencil-and-paper survey distributed to the general in- and outpatient population at this medical center. These surveys, called Spiritual Assessments (SAs), are offered by Chaplains to patients who request Chaplaincy services. The primary aim of a SA is to document that a patient has consented to receiving Chaplaincy services and obtain additional information necessary for providing such services (Department of Veterans Affairs, 2008). SAs were neither originally designed for research purposes nor do they represent a validated survey instrument.

Chaplaincy services are available to all patients, across all clinical departments, without bias to faith affiliation or level of devotion. Chaplains generally provide services in the course of routine visits to the various clinical departments as well as upon request by a patient. Before completing a SA, all patients are informed that filling out the survey is voluntary and that there is no right or wrong way to answer a question. SAs are filled out independently by patients and either returned to or picked up by the Chaplaincy Department after completion. Surgical outpatients visited by a Chaplain are provided a self-addressed envelope for mailing their SA to the medical center following their procedure.

SAs returned to the medical center's Chaplaincy Department are processed and a note made in the patient's medical record. At this stage, a Chaplain could see if a patient had previously filled out an SA and follow up with the Veteran concerning any changes in how they responded to a given question. SAs were also entered into a de-identified database used by Chaplains at the medical center for professional development purposes. Multiple SAs completed by the same individual were so acknowledged in this database. The secondary use of this database for research purposes was granted institutional review board approval by the Research and Development Committee at the Veterans Affairs Medical Center in Syracuse, New York.

This study analyzes SAs returned to the Chaplaincy Department from August 2002 to June 2013. All respondents were either in- or outpatients at the data collection site during this period and needed to have expressed a desire for Chaplaincy services. Patients who may have completed more than one SA during this period only had their first SA analyzed as part of this study. Invalid responses (e.g. more than one answer) were also omitted from the analytic data set.

### Spiritual health

Respondents were asked (Q1) “How would you rate your spiritual health? Are you in … ?” Answer options included (1) excellent spiritual health, (2) good spiritual health, (3) fair spiritual health, (4) poor spiritual health, or (5) uncertain about my spiritual health. The SA did not specify what was to be understood by spiritual health, allowing for individual interpretation by the Veteran. What a Veteran may have understood as spiritual health would have been discussed in private with a Chaplain.

Part of the challenge of applying spirituality and religion to suicide prevention efforts is that spiritual well-being has yet to be operationalized into a standardized construct. Whereas general consensus exists as to the definition of religion (i.e. as a set of beliefs, practices, and rituals), defining spiritual health remains a highly contentious issue, with no agreement as to the characteristics of spirituality (Chuengsatiansup, 2003; Koenig et al., 2012; Kopacz et al., in press). Attempts at defining spiritual well-being have seen it conceptualized as “the central philosophy of life that guides conduct and the meaning-giving center of human life which influences all individual and social behavior” (Moberg, 1979), as a function of religious and existential well-being (Paloutzian & Ellison, 1982), or, more simply, “as a complex and multidimensional part of the human experience – our inner belief system” (Joint Commission on Accreditation of Healthcare Organizations, 2005).

### Perceptual variables

Respondents were also asked two additional perceptual questions related to factors intuitively thought to influence spiritual health. These included (Q2) “How would you classify your spiritual life? Do you see yourself being … ?” Answer options were (1) deeply spiritual, (2) fairly spiritual, (3) only slightly spiritual, (4) not at all spiritual, or (5) against anything spiritual. Respondents were next asked (Q3) “How much is your spiritual life a source of strength and comfort to you?” Answer options included (1) my primary or only source, (2) a great deal, (3) quite a bit, (4) slightly, or (5) not at all/not applicable.

### Suicide ideation

Respondents were also presented with a question listing 31 life events and asked to “Check the box, if in the last two years you have experienced … ” Those who did not check this box were not considered to have experienced suicide ideation. Those who did not check this box were considered to not have experienced suicide ideation.

Suicide ideation may be indicative of an increased risk for suicide. While those who express suicidal ideation alone seldom go on to die by suicide, the association between ideation and suicidal behavior is well established (Gliatto & Rai, 1999; Hall, Platt, & Hall, 1999; Nock et al., 2008; Wilcox et al., 2010). The prevalence estimates of suicidal ideation among Veteran populations range from 6.5% to almost 46%, being more common in Veterans with a psychiatric diagnosis (Corson et al., 2013; Richardson et al., 2012).

### Statistical analysis

In keeping with the cross-sectional design of this study, statistical analyses were largely descriptive, with results being presented as frequencies (Grimes & Schultz, 2002; Tullis & Albert, 2008). Respondents were first divided into two groups based on self-reported suicide ideation. Differences in the distribution of responses to spiritual health (Q1) were examined between these two groups using chi-squared (*χ* ^2^). The distribution of responses to spiritual health was subsequently compared between the two groups based on each of the perceptual variables (Q2 and Q3). Individual responses for spiritual health were paired with a given perceptual variable and any differences in the distribution of responses between ideators and non-ideators were identified using *χ* ^2^.

The perceptual variables were analyzed using a “top- and bottom-box” approach, an accepted means for analyzing self-reported data (Tullis & Albert, 2008). This approach converts Likert-type values into nominal data, sorted according to the highest/best and lowest/worst values in a survey (Velanovich, 2007). In analyzing a five-point Likert-type scale, the top-box most often includes the top two “most positive” responses, whereas the bottom-box usually contains the remaining three “least positive” responses (Allen & Wilburn, 2002). For spiritual health, this included (1) excellent or good and (2) fair or poor or uncertain. How one classifies their spiritual life was dichotomized as (1) deeply or fairly and (2) only slightly or not at all or against anything spiritual. Viewing spirituality as a source of strength and comfort was dichotomized as (1) my primary or only source or a great deal and (2) quite a bit or slightly or not at all/not applicable. The main benefit of top- and bottom-box analysis is that it more appropriately represents respondents’ general attitudes toward a given topic/statement, inclusive of taking into consideration the responses of individuals more or less apt to choose an extreme (i.e. positive or negative) Likert-type response as well as controlling for unequal distance between responses (Bell, 2005; Hazelwood, 1989; Kothari, 2004).

Statistical significance was defined as *p* < .05. All analyses took place using SPSS Version 19.0.

## Results

There were 6198 SAs returned to the Chaplaincy Department for processing. Of these, 820 SAs were excluded from data analysis, representative of multiple SAs completed by the same individuals or as containing invalid responses. The final sample included 5378 unique SAs which were used to examine spiritual health (Q1) as well as how one classifies their spiritual life (Q2). One respondent left Q3 blank, meaning that only 5377 SAs were available to examine this variable. It was not possible to calculate a response rate as no data were available detailing the overall number of patients surveyed, the number of SAs distributed, or the distribution of SAs across clinical departments.

Suicide ideation was reported by 549 (10.20%) respondents, compared to 4829 (89.80%) who did not report suicide ideation. In examining the distribution of responses for spiritual health (Q1), significant differences were noted between ideators and non-ideators (Table 1). Non-ideators significantly more often reported being in excellent (*χ* ^2^(1) = 104.69, *p* < .0001) or good (*χ* ^2^(1) = 86.13, *p* < .0001) spiritual health. Ideators significantly more often reported being in fair (*χ* ^2^(1) = 50.84, *p* < .0001), poor (*χ* ^2^(1) = 175.91, *p* < .0001), or uncertain (*χ* ^2^(1) = 102.79, *p* < .0001) spiritual health.

**Table 1.** How would you rate your spiritual health?

	Ideators (*N* = 549) *N* (%)	Non-ideators (*N* = 4829) *N* (%)	*χ*^2^	*p*-Value
Excellent	30 (5.46)	1198 (24.36)	104.69	<.0001
Good	121 (22.04)	2055 (41.79)	86.13	<.0001
Fair	197 (35.88)	1074 (21.84)	50.84	<.0001
Poor	120 (21.86)	290 (5.90)	175.91	<.0001
Uncertain	81 (14.75)	212 (4.31)	102.79	<.0001

Using top- and bottom-box analysis, significant differences existed for spiritual health based on how one classified their spiritual life (Q2). Among respondents who identified as deeply or fairly spiritual (top-box), non-ideators more often reported being in excellent or good spiritual health, whereas ideators more often reported being in fair or poor or uncertain spiritual health (*χ* ^2^(1) = 250.75, *p* < .0001) (Table 2). Among those who identified as only slightly or not at all or against anything spiritual (bottom-box), non-ideators more often reported being in excellent or good spiritual health, while ideators more often reported being in fair or poor or uncertain health (*χ* ^2^(1) = 16.34, *p* < .0001) (Table 3).

**Table 2.** How would you classify your spiritual life?

Self-rated spiritual health	Ideators (%)	Non-ideators (%)	*χ*^2^	*p*-Value
Excellent OR good	146 (37.44)	3159 (75.11)	250.75	<.0001
Fair OR poor OR uncertain	244 (62.56)	1047 (24.89)		

Note: Respondents who answered deeply OR fairly spiritual (top-box).

**Table 3.** How would you classify your spiritual life?

Self-rated spiritual health	Ideators (%)	Non-ideators (%)	*χ*^2^	*p*-Value
Excellent OR good	5 (3.14)	94 (15.09)	16.34	<.0001
Fair OR poor OR uncertain	154 (96.86)	529 (84.91)		

Note: Respondents who answered only slightly OR not at all OR against anything spiritual (bottom-box).

Significant differences were also found for spiritual health when taking into consideration how respondents saw spirituality as a source of strength and comfort (Q3). Among those who saw spiritual life as their primary or only source or as a great deal of strength and comfort (top-box), non-ideators were more likely to rate their spiritual health as excellent or good, whereas ideators were more likely to rate their spiritual health as fair or poor or uncertain (*χ* ^2^(1) = 196.23, *p* < .0001) (Table 4). For those who saw their spiritual life as being quite a bit or slightly a source of strength and comfort or not at all/not applicable (bottom-box), non-ideators significantly more often rated their spiritual health as excellent or good, whereas ideators rated their spiritual health as fair or poor or uncertain (*χ* ^2^(1) = 61.00, *p* < .0001) (Table 5).

**Table 4.** How much is your spiritual life a source of strength and comfort to you?

Self-rated spiritual health	Ideators (%)	Non-ideators (%)	*χ*^2^	*p*-Value
Excellent OR good	117 (49.37)	2713 (85.10)	196.23	<.0001
Fair OR poor OR uncertain	120 (50.63)	475 (14.90)		

Note: Respondents who answered my primary or only source OR a great deal (top-box).

**Table 5.** How much is your spiritual life a source of strength and comfort to you?

Self-rated spiritual health	Ideators (%)	Non-ideators (%)	*χ*^2^	*p*-Value
Excellent OR good	34 (10.90)	539 (32.87)	61.00	<.0001
Fair OR poor OR uncertain	278 (89.10)	1101 (67.13)		

Note: Respondents who answered quite a bit OR slightly OR N/A (bottom-box).

## Discussion

The aim of this study was to compare the self-rated spiritual health of Veterans with and without suicide ideation. Non-ideators significantly more often described their spiritual health as excellent or good, compared to ideators who more often described their spiritual health as fair, poor, or uncertain. The distribution of responses to spiritual health was further examined in the context of two perceptual variables. Non-ideators were found to more often describe their spiritual health as excellent or good, regardless of how they classified their spiritual life or the extent to which they saw spiritual life as a source of strength and comfort. Regardless of their response to either perceptual variable, ideators would more often describe their spiritual health as fair, poor, or uncertain.

The results suggest that Veterans who reported having endorsed suicide ideation in the two years preceding their SA are in worse self-rated spiritual health than Veterans without suicide ideation. This finding may have several practical implications for suicide prevention research. Nonetheless, at present, any discussion related to examining the place of spirituality in suicide prevention will inevitably yield more questions than answers.

In first order, the observed differences in spiritual health between ideators and non-ideators highlight that spiritual well-being may indeed be relevant to Veteran suicide prevention efforts. Until recently, the idea of spiritual well-being has been largely absent from studies examining suicidal behavior in Veteran population. Future studies should look to better understand how spirituality influences the suicide trajectory of Veterans, including its potential role as a protective factor against suicidal behavior. Alternatively, writes one author, “by ignoring the spiritual dimension of health, for whatever reason, we may be depriving ourselves of the leverage we need to help empower individuals and populations to achieve improved physical, social, and mental health” (Vader, 2006).

Significant differences in spiritual health continued to exist between ideators and non-ideators even when dealing with Veterans who described themselves as generally pious or who ascribed considerable significance to their spiritual lives. This underscores the complex empirical challenge of identifying the factors which contribute to a positive sense of spiritual well-being. What also remains to be determined are factors which may be unique to Veteran populations. More effectively conceptualizing spiritual well-being for the purposes of suicide prevention stands to increase the capacity for practically applying spirituality as part of an organized intervention.

Of note is that Chaplaincy services were provided to some respondents who reported being “not at all spiritual” or “against anything spiritual” (Q2). While it was beyond the scope of this study to qualitatively assess why respondents sought Chaplaincy services, this finding suggests that religious/spiritual support may not be the only motivating factor for meeting with a Chaplain. Research suggests that many Veterans consider Chaplains to be trusted confidants (Hughes & Handzo, 2010; Nieuwsma et al., 2013). In addition to religious/spiritual services, Chaplains also provide such “secular” services as crisis intervention, emotional enabling, ethical consultation-deliberation, life review, patient advocacy, counseling, bereavement, and empathetic listening (Handzo et al., 2008).

It was also beyond the scope of this study to examine how receptive Veterans at risk of suicide might be to a spiritually minded intervention. Despite the potential appeal of such an intervention, it stands to reason that services aimed at alleviating more immediate sources of distress (e.g. housing, healthcare access) would most likely be in greater demand by at-risk populations (Kyle & Dunn, 2008). Spiritual care (e.g. Chaplaincy services) might then serve as a complement to existing support and healthcare options (Nieuwsma et al., 2013). Additional research is needed to better understand the demand/need for spiritual care services among at-risk Veterans. This would allow for properly placing spiritual care within the hierarchy of services provided to this group.

A major limitation of this study was the fact that spiritual health was not precisely defined in the SA. As a result, variability in how respondents understood this construct is to be expected. Identifying a genuine relationship between spirituality and different health outcomes is further made difficult considering the general lack of an empirically validated definition of spiritual health for suicide research. For example, while some respondents may have taken spiritual health to mean something religious or metaphysical or existential, others may have understood it more generally as a psychological or social state, reflecting a sense of meaning, purpose, peace, hope, etc. in their lives (Koenig et al., 2012). Future research should look to establish a definition of spiritual well-being which is appropriate for and reflective of Veteran populations.

The results of this study must also be weighed against other limitations. The sample population represents a sample of convenience, biased toward Veterans with health problems (i.e. seeking healthcare services at the data collection site), who agreed to meet with a Chaplain, and complete a SA. The findings should not, therefore, be generalized to the overall Veteran population or to the general population of Veterans who access VHA healthcare services. As the analytic data set was de-identified, it was neither possible to verify responses related to suicide ideation using the Veteran's medical record nor was it possible to obtain additional data (e.g. demographic, co-morbid conditions, etc.) for more robust data analysis. The cross-sectional scope of the data set, the transient nature of suicide ideation, as well as how suicide ideation was measured (i.e. two years before the SA) could also suggest recall bias. Respondents endorsing suicide ideation upwards of two years ago may have been less likely to recall and/or report the event compared to respondents with current or recent suicide ideation.

Despite these limitations, this study presents exciting opportunities for additional research. The results suggest that spiritual well-being may indeed be relevant to suicide prevention efforts in Veteran populations. As the first study of its kind to quantitatively examine spiritual health in a group of Veterans, those with suicide ideation significantly more often rated their spiritual health as worse than that of Veterans without suicide ideation. Be that as it may, the viability and potential utility of applying spiritual well-being as part of an organized intervention has yet to be determined and should remain the subject of future research.
